# Predictive Value of Occult Metastasis and Survival Significance of Metabolic Tumor Volume Determined by PET-CT in cT1-2N0 Squamous Cell Carcinoma of the Tongue

**DOI:** 10.3389/fonc.2020.542530

**Published:** 2020-12-04

**Authors:** Lijie Yang, Fei Liu, Yao Wu, Qigen Fang, Xiaojun Zhang, Wei Du, Xu Zhang, Defeng Chen, Ruihua Luo

**Affiliations:** ^1^ Department of Stomatology, The First Affiliated Hospital of Zhengzhou University, Zhengzhou, China; ^2^ Department of Head Neck and Thyroid, Affiliated Cancer Hospital of Zhengzhou University, Henan Cancer Hospital, Zhengzhou, China

**Keywords:** PET-CT, metabolic tumor volume, early-stage squamous cell carcinoma of the tongue, oral squamous cell carcinoma, tongue squamous cell carcinoma

## Abstract

**Objectives:**

Our goal was to analyze the possibility of using metabolic tumor volume (MTV) to predict occult cervical metastasis and survival in cT1-2N0 squamous cell carcinoma (SCC) of the tongue.

**Methods:**

Data on the primary tumor MTV and cervical node status as determined by the maximum standardized uptake value were retrieved. The sensitivity and specificity in predicting occult metastasis were calculated with a fourfold table. Associations between occult metastasis and clinicopathological variables were evaluated by univariate and multivariate analyses. The main study endpoints were locoregional control (LRC) and disease-specific survival (DSS).

**Results:**

A total of 24 (20.3%) of 118 patients had occult metastasis. An MTV cutoff value of 4.3 cm^3^ showed a sensitivity of 50.0% and a specificity of 76.6% in predicting occult metastasis. The sensitivity and specificity of PET-CT in predicting occult metastasis in cT1 tumors were 66.6 and 89.8%, respectively, with values of 83.3 and 67.3%, respectively, when combined with the MTV. The sensitivity and specificity of PET-CT in predicting occult metastasis in cT2 tumors were 72.2 and 82.2%, respectively, with values of 88.9 and 57.8%, respectively, when combined with the MTV. Patients with MTV ≥4.3 cm^3^ had a higher occult metastasis rate than patients with MTV <4.3 cm^3^. The 5-year LRC and DSS rates were 86 and 94%, respectively, in patients with MTV <4.3 cm^3^ and 54 and 72%, respectively, in patients with MTV ≥4.3 cm^3^. Both differences were found to be significant in univariate and multivariate analyses.

**Conclusions:**

MTV ≥4.3 cm^3^ was associated with an increased probability of occult metastasis and lower LRC and DSS rates in early-stage SCC of the tongue.

## Introduction

Squamous cell carcinoma (SCC) of the tongue is the most common malignancy in the oral cavity, and surgical excision is the mainstay treatment (1). However, whether elective neck dissection (END) should be included in the treatment of early-stage lesions (cT1-2N0) of the tongue remains controversial. The main argument supporting routine END is the early detection of occult metastasis, which allows for adjustment of the adjuvant treatment plan and improvement in prognosis (2, 3).

However, the reported occult metastasis rate varies based on race and geographical differences (4–7), and a few authors have noted that a wait-and-see policy does not compromise survival (4, 5). Further, the shoulder pain and dysfunction associated with neck dissection, although uncommon, should not be neglected (8).

The lack of reliable predictors for occult metastasis contributes to the discussion. Although perineural invasion (PNI), lymphovascular invasion (LVI), depth of invasion (DOI), extranodal extension (ENE), and lingual lymph node metastasis have been shown to increase the rate of occult metastasis (1, 9, 10), these variables are always unknown prior to the availability of definite postoperative pathology, and their clinical application is greatly limited.

PET-CT has been widely used for primary tumor staging and nodal metastasis detection (11), and the metabolic tumor volume (MTV), a PET-CT parameter, has been extensively analyzed regarding its prognostic role in head and neck SCC (12–15). However, the head and neck include multiple anatomical areas, and the characteristics of SCC of the tongue differ from those of cancers arising in other areas. Additionally, very few authors have evaluated the association between MTV and occult metastasis in cT1-2N0 tongue SCC. Therefore, we herein aimed to analyze the reliability of the MTV in predicting occult lymph node metastasis and the prognostic role of MTV in early-stage SCC of the tongue.

## Patients and Methods

### Ethical Considerations

The Zhengzhou University Institutional Research Committee approved our study, and all patients signed informed consent agreements for medical research before the initial treatment. All procedures were performed in accordance with the relevant guidelines and regulations.

### Patient Selection

The medical records of patients with primary cT1-2N0 SCC of the tongue treated with surgery from January 2010 to January 2015 were retrospectively reviewed. The inclusion criteria were as follows: no history of another malignancy; performance of preoperative PET-CT; at least 5 years of follow-up data available. Patient information regarding demographics, smoking, drinking, pathology, MTV, and follow-up was extracted and analyzed.

### Important Variable Definitions

cT1-2 was defined as tumors with a diameter of less than 4 cm and a DOI of less than 10 mm on CT/MRI according to the 8^th^ American Joint Committee on Cancer (AJCC) classification. Patients had no evidence of positive lymph nodes at palpation and at the preoperative clinical examination. A lymph node was defined as positive on ultrasound if the smallest diameter was ≥10 mm, the ratio of the longest to smallest diameter was ≤2, and changes occurred in the internal anatomical structure of lymph nodes. The findings suggesting node positivity on CT or MRI were the following: area with clear evidence of non-fat, low-density, or liquid components; largest diameter >15 mm at level II and >10 mm at other levels; and ratio of the longest to smallest diameter ≤2. Drinkers were defined as those who consumed at least one alcoholic drink per day for at least 1 year, and smokers were defined as those who smoked on a daily basis or had quit smoking less than 5 years prior to the study (1, 16, 17). PNI was considered to be present if tumor cells were identified within the perineural space and/or nerve bundle; LVI was considered positive if tumor cells were noted within the lymphovascular channels (1, 11, 16, 17). Pathological DOI was measured from the level of the adjacent normal mucosa to the deepest point of tumor infiltration regardless of the presence or absence of ulceration (18). ENE was considered present if tumor cells were noted outside the capsule of a metastatic lymph node (19).

### Treatment Plan

At our cancer center, systemic examinations, including ultrasound, CT, and MRI, were routinely performed for every patient with tongue SCC, while PET-CT was selectively performed. All patients underwent complete resection of the primary tumor with a margin of at least 1 cm. If necessary, a free flap or pedicled flap was used to close the defect, and selective neck dissection (I–IV) was routinely performed.

### Specimen Processing

The primary lesion and cervical lymph nodes were sent for pathological analysis separately, and the cervical specimens were divided into four subgroups based on the neck level. All specimens were serially sectioned at a thickness of 5-mm at an interval of 0.5 mm and stained with hematoxylin and eosin. Immunohistochemistry was performed as needed to make an accurate pathological diagnosis.

### Follow-Up

After being discharged from the hospital, the patients were followed-up every 3 months for the first two years, every 6 months from the third to fifth year, and once a year thereafter.

### PET-CT Scheme

Several PET-CT scanners were used to perform PET-CT scans (GE Healthcare, Milwaukee, America). The patients fasted for at least 6 h before the PET-CT scan, and the procedure was postponed if the glucose level was >200 mg/dl. Each patient received 10–20 mCi of [^18^F] FDG dosed based on his or her weight. Axial PET and diagnostic CT images were obtained from the calvarial vertex through the upper thighs after urinary voiding. Emission images were obtained 60 min after radiopharmaceutical injection. No contrast medium was used during the CT scan. The images were obtained at a slice thickness of 2.5 mm and reconstructed. The maximum standardized uptake (SUVmax) value was measured for both the primary tumor and regional lymph nodes. For every suspicious lesion, the isocontour region of interest centered on the maximum-value pixel was drawn automatically using workstation tools to generate the SUVmax of the region. An SUVmax cutoff of 2.5 MBq/g was used for FDG-avid lymph nodes and primary tumors on PET-CT. A nuclear medicine doctor with 10 years of experience calculated the MTVs of the primary tumors using the attenuation-corrected PET data and a commercial software package (Advantage Workstation VolumeShare version 2; GE Health).

### Statistical Analysis

The last follow-up was performed between January 2020 and February 2020 to ensure that the minimum follow-up time was longer than 5 years. The MTV cutoff value for the primary tumor was calculated by receiver operating characteristic (ROC) curve analysis. The sensitivity and specificity of the nodal status as determined by the SUVmax and MTV alone in predicting occult metastasis were calculated using the pathological outcome as the reference standard, and a parallel test was used when combining the nodal status as determined by the SUVmax and MTV. The result was defined as positive if either the nodal status as determined by the SUVmax or the MTV suggested the presence of lymph node metastasis; the result was defined as negative if neither the SUVmax nor the MTV supported the presence of lymph node metastasis. Associations between occult nodal metastasis and clinicopathological variables were evaluated by the chi-square test. Factors that were significant according to the chi-square test were then analyzed by multivariate linear regression to determine independent factors. The study endpoints were locoregional control (LRC) and disease-specific survival (DSS). The duration of LRC was calculated from the date of surgery to the date of the first locoregional recurrence or the last follow-up. The duration of DSS was calculated from the date of surgery to the date of cancer-related death or the last follow-up. The Kaplan-Meier method was used to analyze the LRC and DSS rates, and the factors that were significant on univariate analysis were then evaluated by the Cox model to determine the independent factors. Statistical analyses were performed using SPSS 20.0. All reported p values were two-sided, and p < 0.05 was considered significant.

## Results

### Demographic and Pathological Data

A total of 118 patients (80 males and 38 females) were included in the analysis. The mean age was 57.6 (range: 32–78) years. Smoking and drinking were noted in 85 (72.0%) and 66 (55.9%) patients, respectively.

The clinical tumor stage was cT1 in 55 (46.6%) patients and cT2 in 63 (53.3%) patients. PNI and LVI were present in 19 (16.1%) and 15 (12.7%) patients, respectively. The mean DOI was 4.2 mm, with a range from 1.0 to 7.5 mm. Tumors were characterized as well-differentiated in 32 (27.1%) patients, moderately differentiated in 47 (39.8%) patients, and poorly differentiated in 39 (33.0%) patients. There were no cases of ENE. Fifteen (12.7%) patients underwent reconstruction with a radial forearm flap, and 10 (8.5%) patients underwent reconstruction with a submental island flap. Negative margins were achieved in all patients.

### ROC Curve and PET-CT Evaluations

**Figure 1:**
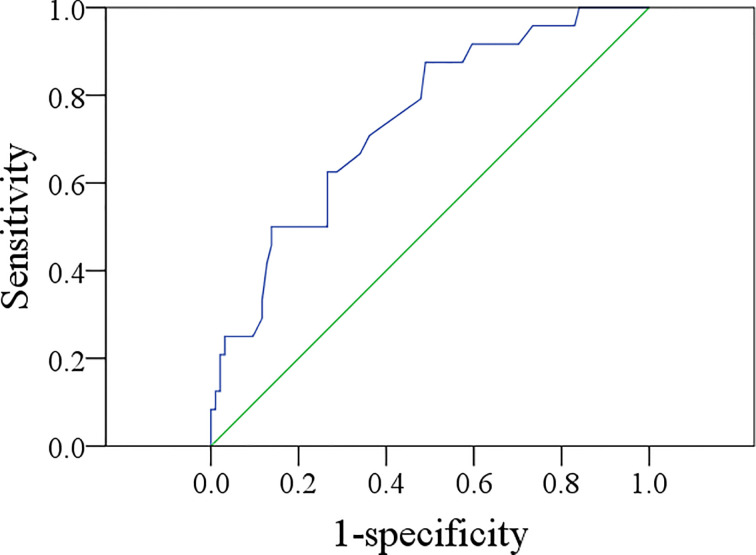
ROC curve of metabolic tumor volume in predicting occult metastasis (AUC = 0.740).

The mean MTV was 3.3 cm^3^, with a range from 0.6 to 8.4 cm^3^. The ROC curve analysis showed that the best MTV cutoff value was 4.3 cm^3^, with a sensitivity of 50.0% and a specificity of 76.6% in predicting occult metastasis (AUC = 0.740) (**Figure 1**).

Among patients with cT1 tumors, nine showed positive neck lymph node disease on PET-CT, four of whom exhibited metastasis upon pathological examination, and 46 were negative on the neck, two of whom exhibited metastasis upon pathological examination; the sensitivity and specificity of PET-CT in predicting occult metastasis were 66.6 and 89.8%, respectively. When combining the nodal status and MTV by PET-CT, the sensitivity and specificity were 83.3 and 67.3%, respectively.

Among patients with cT2 tumors, 21 showed positive neck lymph node disease on PET-CT, 13 of whom exhibited metastasis upon pathological examination; 42 patients were negative on the neck, five of whom exhibited metastasis upon pathological examination; the sensitivity and specificity of PET-CT in predicting occult metastasis were 72.2 and 82.2%, respectively. When combining the nodal status and MTV by PET-CT, the sensitivity and specificity were 88.9 and 57.8%, respectively.

### Potential Predictors for Occult Metastasis

Cervical metastasis occurred in 24 (20.3%) patients. Among them, six patients had a cT1 tumor, with an occult metastasis rate of 10.9%, and 18 patients had a cT2 tumor, with an occult metastasis rate of 28.6%; the difference was significant (p = 0.017). Among patients with MTV ≥4.3 cm^3^, 12 (35.3%, 12/34) had occult metastasis; among patients with MTV <4.3 cm^3^, 12 (14.3%, 12/84) had occult metastasis, and the difference was significant (p = 0.010). Among patients with a DOI >5 mm, 15 (30.0%, 15/50) had occult metastasis; among patients with a DOI ≤5 mm, nine (13.2%, 9/68) had occult metastasis, and the difference was significant (p = 0.025). Among patients with a poorly differentiated tumor, 14 (35.9%, 14/39) had occult metastasis; among patients with a well-differentiated or moderately differentiated tumor, 10 (12.7%, 10/79) had occult metastasis, and the difference was significant (p = 0.003). There were no significant associations between occult metastasis and other clinicopathological variables (**Table 1**). On further multivariate analysis, the risk of occult metastasis was increased by 3.2-fold in the case of a DOI >5 mm, by 2.7-fold in the case of a cT2 tumor, and by 1.8-fold in the case of MTV ≥4.3 cm^3^ (**Table 2**).

**Table 1:** Univariate analysis of potential predictors for occult metastasis in the 118 patients.

Variables	Occult metastasis		
	Positive (n = 24)	Negative (n = 94)	p
Age			
<40	3	7	
≥40	21	87	0.685
Sex			
Male	19	61	
Female	5	33	0.182
Smoker			
Presence	20	65	
Absent	4	29	0.167
Drinker			
Presence	13	53	
Absent	11	41	0.845
Metabolic tumor volume (cm^3^)			
<4.3	12	72	
≥4.3	12	22	0.010
Clinical tumor stage			
T1	6	49	
T2	18	45	0.017
Depth of invasion			
>5 mm	15	35	
≤5 mm	9	59	0.025
Perineural invasion			
Presence	5	14	
Absent	19	80	0.480
Lymphovascular invasion			
Presence	5	10	
Absent	19	84	0.181
Tumor differentiation			
Well + moderate	10	69	
Poor	14	25	0.003

**Table 2:** Multivariate analysis of potential predictors for occult metastasis in the 118 patient.

Variables	p	HR (95% CI)
Metabolic tumor volume ≥4.3 cm^3^	0.023	1.8 (1.245–3.531)
cT2	0.002	2.7 (1.334–5.483)
Depth of invasion >5 mm	0.001	3.2 (1.467–6.826)
Poor tumor differentiation	0.067	2.5 (0.964–7.443)

### Survival Analysis

**Figure 2:**
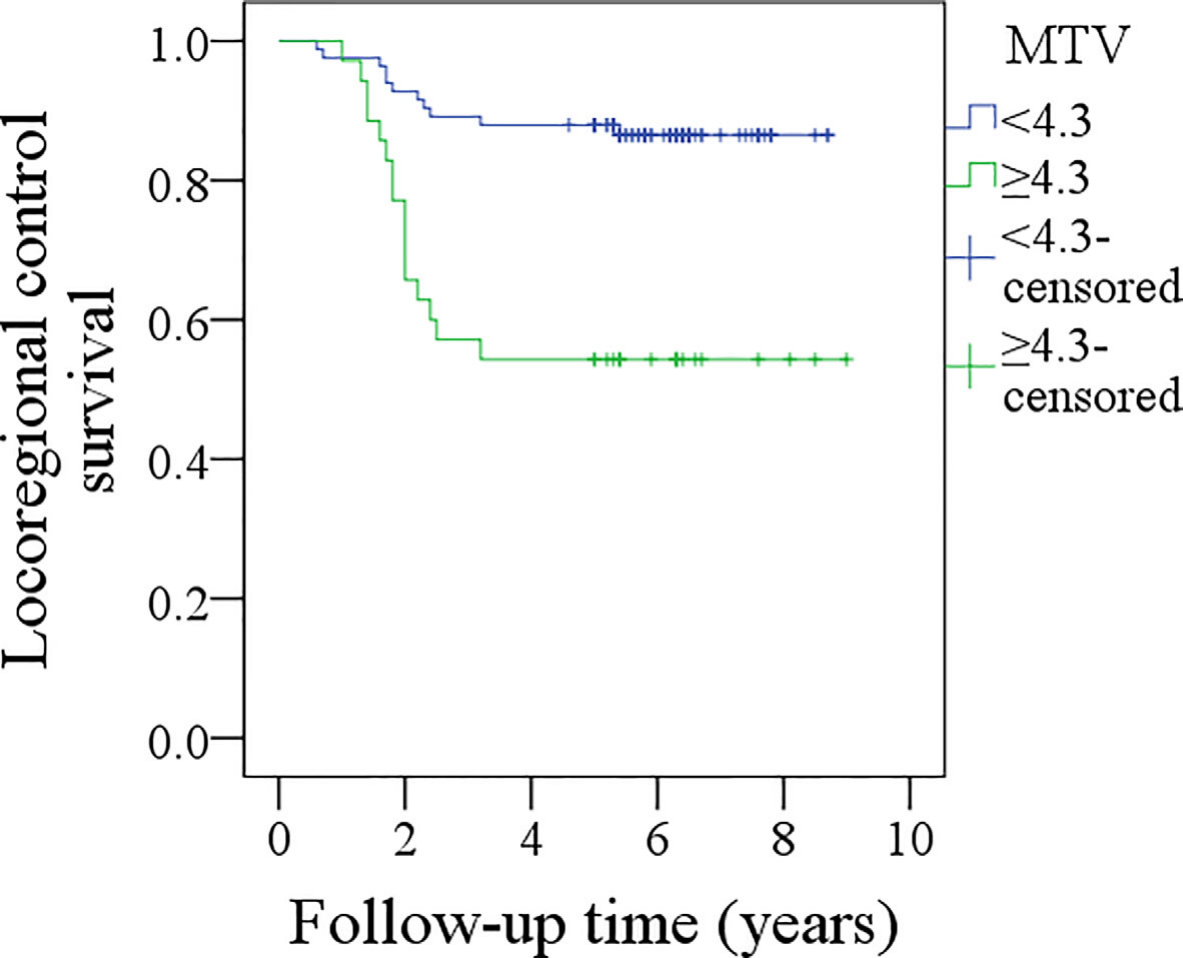
Comparison of locoregional control survival between patients with different metabolic tumor volume (MTV) (p < 0.001).

During the follow-up period, with a median of 5.3 years, 30 patients received adjuvant radiotherapy, and four patients also received adjuvant chemotherapy. Locoregional recurrence occurred in 27 patients, there were no cases of distant metastasis, and 15 patients died of the disease. The overall 5-year LRC and DSS rates were 77 and 88%, respectively. In patients with MTV<4.3 cm^3^, the 5-year LRC rate was 86%, and in patients with MTV≥4.3 cm^3^, the 5-year LRC rate was 54%; the difference was significant (p < 0.001) (**Figure 2**). Further analysis using the Cox model indicated the independent influence of MTV ≥4.3 cm^3^ in decreasing the LRC rate (**Table 3**).

**Table 3:** Univariate and Cox model analyses for locoregional control survival in the 118 patients.

Variable	Univariate	Cox model	
	p	p	HR (95% CI)
Age (<40 *vs* ≥40)	0.324		
Sex (Male *vs* female)	0.547		
Smoker	0.078		
Drinker	0.432		
Metabolic tumor volume (≥4.3 *vs <*4.3)	<0.001	0.003	2.565 (1.264–6.449)
Clinical tumor stage	<0.001	0.002	2.876 (1.462–7.038)
Depth of invasion	<0.001	<0.001	3.003 (1.228–7.663)
Perineural invasion	0.023	0.013	1.889 (1.264–4.775)
Lymphovascular invasion	0.067		
Tumor differentiation (Poor *vs* others)	0.012	0.007	2.722 (1.369–6.418)
Adjuvant treatment	0.226		

**Figure 3:**
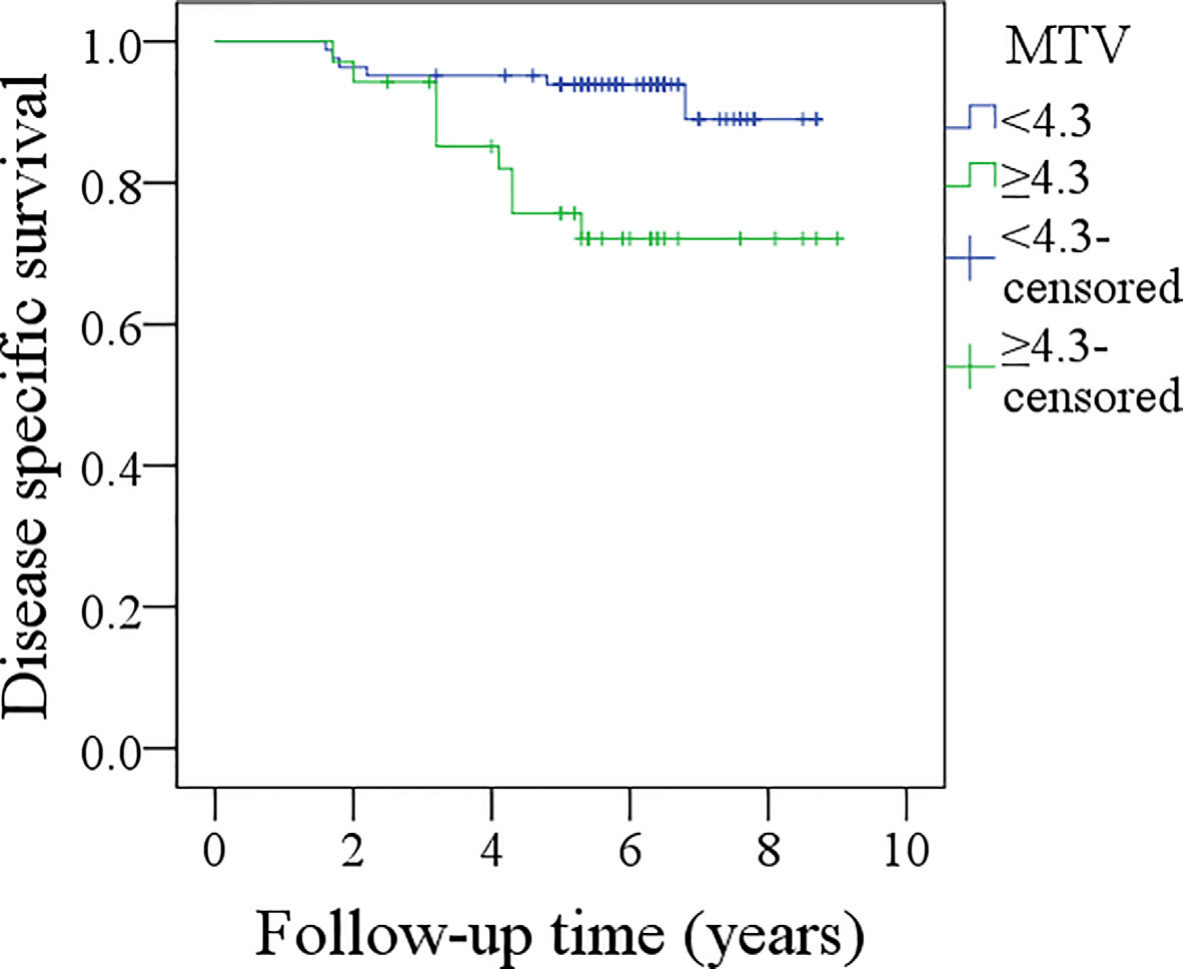
Comparison of disease specific survival between patients with different metabolic tumor volume (MTV) (p = 0.004).

In patients with MTV <4.3 cm^3^, the 5-year DSS rate was 94%, and in patients with MTV≥4.3 cm^3^, the 5-year DSS rate was 72%; the difference was significant (p = 0.004, **Figure 3**). Further analysis using the Cox model indicated the independent influence of MTV ≥4.3 cm^3^ in decreasing the DSS rate (**Table 4**).

**Table 4:** Univariate and Cox model analyses for disease specific survival in the 118 patients.

Variable	Univariate	Cox model	
	p	p	HR (95%CI)
Age (<40 *vs* ≥40)	0.065		
Sex (Male *vs* female)	0.123		
Smoker	0.078		
Drinker	0.431		
Metabolic tumor volume (≥4.3 *vs <*4.3)	0.004	0.003	2.654 (1.447–7.429)
Clinical tumor stage	<0.001	0.001	2.987 (1.628–9.337)
Depth of invasion	<0.001	<0.001	3.943 (1.773–8.783)
Perineural invasion	0.087		
Lymphovascular invasion	0.004	0.034	1.897 (1.243–4.227)
Tumor differentiation (Poor *vs* others)	0.007	0.019	2.001 (1.482–4.836)
Adjuvant treatment	0.386		

## Discussion

The most significant finding in the current study was that MTV ≥4.3 cm^3^ was associated with a nearly twofold increased risk for occult metastasis, and the sensitivity in predicting occult metastasis increased when the MTV and nodal status were combined by PET-CT with a parallel test. This finding provides a new method to identify patients at high or low risk for cervical nodal metastasis.

The neck lymph node status is the most important prognostic factor; survival is usually decreased by half with even one positive lymph node (1). Therefore, neck dissection is an essential component of primary surgery; however, impairment of strength and flexibility of the neck and shoulder following neck dissection cannot be ignored (20). Substantial controversy still exists regarding cN0 neck management in cT1-2 oral SCC because the reported occult metastasis rate varies greatly, and it is generally stated that END is needed if the occult metastasis rate is higher than 20% (21). In our study, the metastasis rate in patients with cT1 tumors was just 10.9%; therefore, the motivation to perform END might be questioned. However, some high-quality studies in the literature support routine neck dissection in cT1-2N0 oral SCC. D’Cruz et al. (22) compared the oncologic outcomes between the elective and therapeutic surgery groups in a prospective, randomized, controlled trial and found that END resulted in significantly higher overall survival and DFS rates. Similar findings for patients with small tumors were also reported by Ren et al. (23) and Hutchison et al. (24). The improved survival benefit in the elective surgery group might be explained by the delayed treatment of metastatic neck cancer in patients initially treated with only primary tumor excision. Therefore, it is reasonable to suggest that if patients with metastatic disease could be preoperatively identified by reliable indicators and receive definite treatment, the prognoses might be similar in the two groups. Numerous authors have tried to explore potential predictors, including tumor budding (25), activin A (26), tumor thickness (27), muscular infiltration and vascular embolization (28), and DOI (29). However, these data cannot always be obtained preoperatively, and other reliable and easy-to-access variables must be identified (**Table 5**).

**Table 5:** Demography and pathologic information of the 118 patients.

Variables	Number (%)
Age	
<40	10 (8.5%)
≥40	108 (91.5%)
Sex	
Male	80 (67.8%)
Female	38 (32.2%)
Smoker	85 (72.0%)
Drinker	66 (55.9%)
Metabolic tumor volume (cm^3^)	
<4.3	84 (71.2%)
≥4.3	34 (28.8%)
Clinical tumor stage	
T1	55 (46.6%)
T2	63 (53.4%)
Depth of invasion	
>5 mm	50 (42.4%)
≤5 mm	68 (57.6%)
Perineural invasion	19 (16.1%)
Lymphovascular invasion	15 (12.7%)
Tumor differentiation	
Well	32 (27.1%)
Moderate	47 (39.8%)
Poor	39 (33.0%)
Cervical nodal status	
N0	94 (79.7%)
N+	24 (20.3%)

PET-CT is a non-invasive method that can be used to evaluate a cN0 neck. Ng et al. (30) reported that the sensitivity of PET-CT in predicting occult metastasis was 51.4% based on 134 patients with oral SCC; the PET-CT findings were negative in 108 patients, with true- and false-negative findings in 91 and 17 cases, respectively. Most of those 91 patients had T1/T2 tumors. Zhang et al. (31) retrospectively analyzed 148 patients with cT1-2N0 oral SCC, revealing that the overall rate of occult metastasis was 13.5%, and the sensitivity of PET-CT was only 21.4%; however, the sensitivity slightly increased with a tumor size ≥2 cm and a DOI ≥4 mm. Similarly, the Memorial Sloan-Kettering Cancer Center also showed that among 142 nodal levels in 31 patients treated with neck dissection, nine were pathologically involved, six showed true-positive PET findings, three showed false-negative PET findings, 127 showed true-negative PET findings, and six showed false-positive PET findings, with a sensitivity of 66.7% (32). Other similar results have also been reported by Kim et al. (33) and Kyzas et al. (34). All these results suggest that the clinical application of PET/CT in assessing a cN0 neck is limited by a relatively high number of false-positive cases and low sensitivity for small metastatic deposits based on the SUV. Additional parameters are needed to increase the reliability of this method.

Tumor volume is an important concept, as it varies even among tumors of the same stage because of the wide range of diameters and DOIs within a single tumor stage. The tumor volume might provide more information than the tumor stage and predict nodal metastasis more accurately (35); however, the pathological tumor volume cannot be calculated preoperatively. Current studies in the literature have thus paid more attention to the tumor volume determined by PET-CT, called MTV. However, only two studies have focused on the association between MTV and occult nodal disease. Chung et al. (36) enrolled 43 patients with cT1-2N0 tongue SCC, all of whom had undergone PET-CT preoperatively; occult metastasis was observed in four of the 29 patients with an MTV ≤6 ml and in eight of the 14 patients with an MTV >6 ml, yielding a sensitivity of 66.7% and a specificity of 80.6%. Another study by Ryu et al. (36) include 53 patients with oral SCC with a cN0 neck; occult metastasis was found in 15 of the 30 patients with an MTV >3 ml and in four of the 23 patients with an MTV ≤3 ml, yielding a sensitivity of 78.9% and a specificity of 55.9%. Similarly, in our study, the MTV showed a sensitivity of 50.0% and a specificity of 76.6% in predicting occult metastasis. Although these three studies reported the independent influence of a higher MTV in increasing the risk for occult metastasis, the relatively low sensitivity is of great concern, as it is insufficient for selecting neck management strategy. The current study is the first to report a high sensitivity in predicting occult metastasis when combining MTV and nodal status by PET-CT, even in patients with cT1 tumors. The finding is very interesting and might be useful for guiding neck management. While the role of lymph node MTV is worthy of investigation, this value was difficult to calculate for patients with negative PET-CT results.

The role of MTV in the survival of patients with head and neck SCC has been widely discussed. Choi et al. (13) previously reported that MTV was one of the strongest prognostic factors among several PET-CT parameters and that MTV >8.8 ml was associated with a >5-fold increased risk of tumor progression and mortality after salvage surgery for the treatment of recurrent oral SCC. In a study analyzing HPV-associated oropharyngeal cancer by Floberg et al. (14), the authors noted that MTV >24 ml remained significantly associated with freedom from recurrence, freedom from distant metastasis, and overall survival despite the use of different treatment methods. Similar findings have also been reported by Alluri et al. (12) and Chung et al. (36); moreover, Chung et al. (37) were the only researchers to have analyzed the prognostic significance of the MTV in early-stage SCC of the tongue, but their sample size was small, and the follow-up time was very short. Our oncologic outcomes show that MTV ≥4.3 cm^3^ was associated with lower LRC and DSS rates over at least a 5-year follow-up, which supports previous findings with stronger evidence. Interestingly, since both tumor stage and DOI were independent prognostic factors, the hazard ratio (HR) of tumor volume should have been higher than that of tumor stage and DOI, but the MTV had an even lower HR than the cT2 stage and DOI in the current study. One possible explanation is that the MTV was calculated by the volume of the tumor showing FDG uptake and that this volume was significantly smaller than the actual tumor volume.

Limitations of the current study must be acknowledged: the retrospective nature; not all early-stage patients underwent PET-CT examination in the period of enrollment, the sample size was small. Therefore, before our findings can be transferred into clinical application, a high-quality randomized controlled trial (RCT) is needed. Although two high-quality studies (22, 24) have shown the superiority of an elective treatment of the neck compared to a wait-and-see policy and one RCT is ongoing (38), none of them included PET-CT in patient work up. Based on the relatively high reliability of PET-CT in predicting lymph node metastasis (12, 13, 31–37) and our results, a RCT enrolling patients with cT1-2N0 SCC of the tongue who are assessed by PET-CT would be highly recommended.

In summary, MTV ≥4.3 cm^3^ was associated with an increased probability of occult metastasis and lower LRC and DSS rates in early-stage tongue SCC, and the MTV and nodal status showed high sensitivity and specificity in predicting occult metastasis when combined by PET-CT.

## Data Availability Statement

All datasets generated for this study are included in the article/supplementary files.

## Ethics Statement

The Zhengzhou University institutional research committee approved our study, and all participants signed an informed consent agreement for medical research before initial treatment, and all the related procedures were consistent with Ethics Committee regulations.

## Author Contributions

All the authors contributed to the study design, manuscript writing, study selection, data analysis, study quality evaluation, and manuscript revision. All authors contributed to the article and approved the submitted version.

## Conflict of Interest

The authors declare that the research was conducted in the absence of any commercial or financial relationships that could be construed as a potential conflict of interest.
